# The EP300, KDM5A, KDM6A and KDM6B Chromatin Regulators Cooperate with KLF4 in the Transcriptional Activation of POU5F1

**DOI:** 10.1371/journal.pone.0052556

**Published:** 2012-12-18

**Authors:** Wan-Ping Wang, Tsai-Yu Tzeng, Jing-Ya Wang, Don-Ching Lee, Yu-Hsiang Lin, Pei-Chun Wu, Yen-Po Chen, Ing-Ming Chiu, Ya-Hui Chi

**Affiliations:** 1 Institute of Cellular and System Medicine, National Health Research Institutes, Zhunan, Taiwan; 2 VYM Genome Research Center, National Yang-Ming University, Taipei, Taiwan; 3 Institute of Biotechnology, National Tsing Hua University, Hsinchu, Taiwan; 4 Graduate Institute of Basic Medical Science, China Medical University, Taichung, Taiwan; Baylor College of Medicine, United States of America

## Abstract

POU5F1 is essential for maintaining pluripotency in embryonic stem cells (ESCs). It has been reported that the constitutive activation of *POU5F1* is sustained by the core transcriptional regulatory circuitry in ESCs; however, the means by which *POU5F1* is epigenetically regulated remains enigmatic. In this study a fluorescence-based reporter system was used to monitor the interplay of 5 reprogramming-associated TFs and 17 chromatin regulators in the transcription of *POU5F1*. We show the existence of a stoichiometric effect for SOX2, POU5F1, NANOG, MYC and KLF4, in regulating *POU5F1* transcription. Chromatin regulators EP300, KDM5A, KDM6A and KDM6B cooperate with KLF4 in promoting the transcription of *POU5F1*. Moreover, inhibiting HDAC activities induced the expression of *Pou5f1* in mouse neural stem cells (NSCs) in a spatial- and temporal- dependent manner. Quantitative chromatin immunoprecipitation-PCR (ChIP-qPCR) shows that treatment with valproic acid (VPA) increases the recruitment of Kdm5a and Kdm6a to proximal promoter (PP) and proximal enhancer (PE) of *Pou5f1* whereas enrichment of Ep300 and Kdm6b was seen in PP but not PE of *Pou5f1* promoter. These findings reveal the interplay between the chromatin regulators and histone modifications in the expression of *POU5F1*.

## Introduction

OCT4, also known as POU5F1 (POU domain, class 5, transcription factor 1), is a transcription factor crucial to self-renewal in embryonic stem cells (ESCs) [1]. It is a prerequisite in the production of induced pluripotent stem cells (iPSCs) and needs to be supplemented exogenously [2]–[6]. *POU5F1* is expressed in abundance in mammalian ESCs, but diminishes with the differentiation of the cells into somatic lineages [1]. *POU5F1*, in conjunction with pluripotency-associated transcription factors SOX2 and NANOG, have been shown to be the main regulators in the core transcriptional regulatory circuitry of ESCs [7].

Gene expression is controlled not only by the availability of transcription factors (TFs), but also by the chromatin content. Chromatin is composed of transcriptionally permissive, less condensed euchromatin, and highly condensed and often transcriptionally silenced heterochromatin, both of which correlate with a specific set of epigenetic modifications [8], [9]. A growing body of evidence suggests that epigenetic modifications are necessary for nuclear reprogramming and stem cell differentiation [10]. Histone deacetylase inhibitors (HDACi), such as valproic acid (VPA), trichostatin A (TSA), sodium butyrate (SB), the EHMT2 histone methyltransferase inhibitor BIX-01294, and the DNA methyltransferase inhibitor RG108, have all been shown to enhance efficiency in generating iPSCs [11]–[13], suggesting that crosstalk between TFs and chromatin regulators is required for induced pluripotency.

The expression of POU5F1 is essential for the induction and maintenance of pluripotency, consequently there have been extensive studies regarding the regulatory mechanism involved in the transcription of *POU5F1* [14]–[17]. However, the means by which TFs communicate with chromatin regulators in the expression of *POU5F1* remains to be elucidated. In this study we employed a fluorescence-based reporter assay in conjunction with high-content cell imaging to monitor the interplay of five reprogramming-associated TFs (POU5F1, SOX2, NANOG, KLF4 and MYC) [5] with 17 chromatin regulators, including histone acetyltransferases, methyltransferases, and demethylases [18], for transcription activity in the 5.0 kb upstream region of human *POU5F1* [4]. Our results may lead to new techniques in the field of pluripotency generation and maintenance.

## Methods

### Plasmids

**Figure 1:**
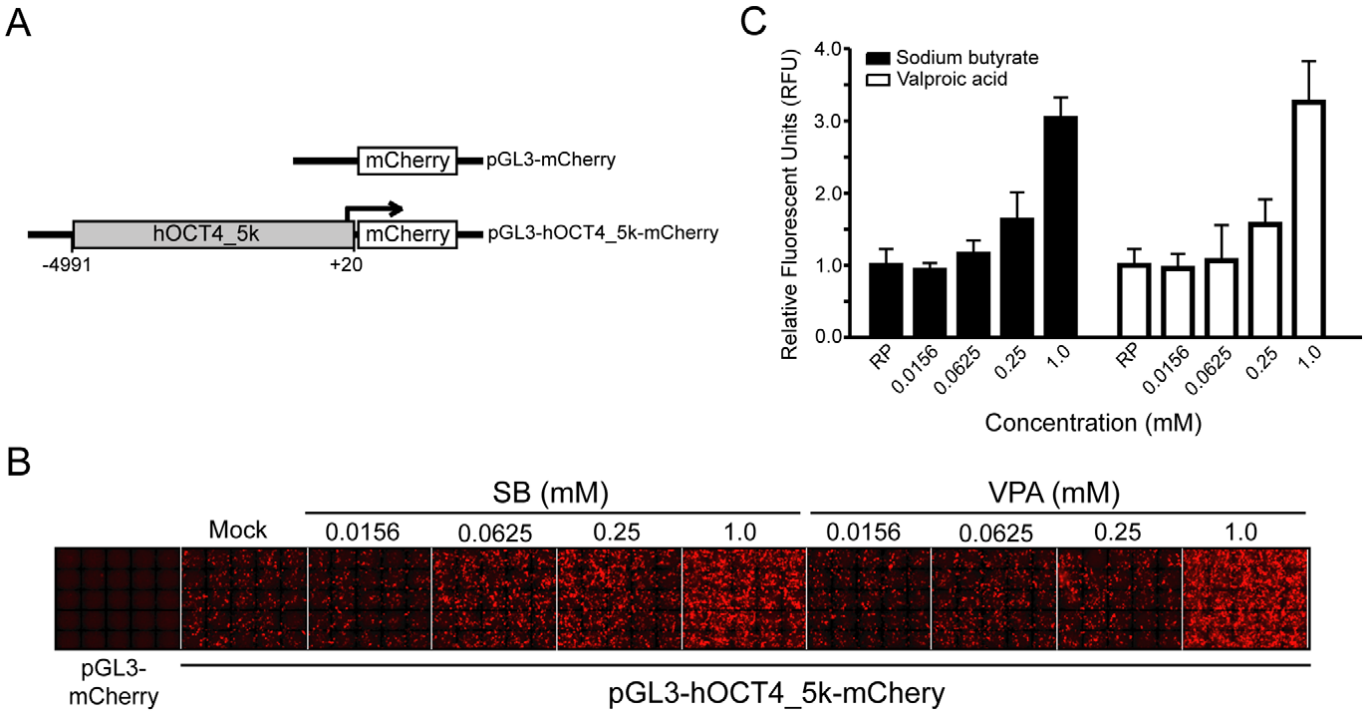
Induction of *POU5F1* transcription by HDAC inhibitors. (A) Scheme presentation of human *POU5F1* proximal 5.0 k upstream region (−4991∼+20) constructed at 5′ end of the *mCherry* fluorescent reporter in vector pGL3 (i.e. pGL3-hOCT4_5 k-mCherry). A plasmid pGL3-mCherry, inserted with *mCherry* but lacking a promoter sequence, was used as a negative control. (B-C) Fluorescence images of HEK293T cells transfected with pGL3-mCherry or pGL3-hOCT4_5 k-mCherry for 24 hours and treated with various concentrations of sodium butyrate (SB) or valproic acid (VPA) for another 24 hours. A dose-dependent increase of mCherry expression was observed and the fluorescent intensity was quantified in (C). RP, pGL3-hOCT4_5k-mCherry reporter only.

The fluorescent protein mCherry-tagged *POU5F1* reporter plasmid (pGL3-hOCT4_5 k-mCherry) was constructed by replacing *Luc* gene in the phOCT4-Luc plasmid (obtained from Addgene, plasmid 17221) [4] that harbors a 5.0 kb upstream region (−4991∼+20) of human *POU5F1* with *mCherry* (Figure 1A). Expression vectors of CREBBP (i.e. CBP, Addgene plasmid 16701) [19], MLL2 (Addgene plasmid 11017) [20], DPY30 (Addgene plasmid 15554) [21], ASH2L (Addgene plasmid 15548) [21], KDM5A (also named RBP2, Addgene plasmid 14800) [22], KDM6A (also named UTX, Addgene plasmid 17438) [23] and KDM6B (also named JMJD3, Addgene plasmid 17440) [23] were obtained from Addgene. The expression vectors of human SOX2, POU5F1, NANOG, MYC, KLF4, KAT2A (also named GCN5L2), KAT8 (also named MYST1), KAT7 (also named MYST2), KDM3A (also named JMJD1A), KDM4A (also named JMJD2A), KDM4C (also named JMJD2C), SUV39H1, SUV39H2 and EHMT2 were obtained by cloning the corresponding complementary DNA (cDNA) into the pCDNA3.0 vector (Invitrogen) and tagged with FLAG at the N-terminus. The EP300 (also named P300) expression plasmid [24] is a generous gift from Kuan-Teh Jeang, National Institute of Allergy and Infectious Disease, the National Institutes of Health (NIH).

### Cell Culture and transfection

Human embryonic kidney 293T (HEK293T) cells were obtained from American Type Culture Collection (ATCC) and maintained in high glucose Dulbecco's Modified Eagle Medium (DMEM, Invitrogen) supplemented with 10% fetal bovine serum (FBS, Biological Industries), 2 mM L-glutamine and antibiotics. PolyJet (SignaGen Laboratories) DNA *in vitro* transfection reagent was used for plasmid transfection. Mouse neural stem cells (mNSCs) were cultured from mouse (strain C57BL/6) embryonic brain as described by Lee et al. [25] with some modifications. In brief, E11.5 mouse brain was minced into pieces and tissues were dissociated with 1 mg/mL hyaluronidase (Sigma-Aldrich) and collagenase IV (Invitrogen) in PBS at 37°C for 5****minutes. After 3 minutes centrifugation at 600×g, cell pellets were further dissociated by Accutase (Millipore) at 37°C for 5 minutes. The dissociated cells were filtered through a sterile nylon mesh (70 μm; BD Bioscience) to obtain single-cell suspension. Cells were pelleted down and resuspended in DMEM/F-12 basal medium containing N2 supplement (Invitrogen), 20 ng/mL epidermal growth factor (EGF, Peprotech), 20 ng/mL basic fibroblast growth factor (bFGF, Peprotech) and 2 μg/mL heparin (Sigma-Aldrich) and incubated in a humidified CO_2_ chamber at 37°C. To induce formation of neurospheres, the cultured neural stem cells were dissociated by Accutase, pelleted down, resuspended in DMEM/F-12 basal medium containing B27 supplement (Invitrogen), 20 ng/mL EGF, 20 ng/mL bFGF and 2 μg/mL heparin and cultured for 7 days. The procedure and use of the animals were approved (protocol number: NHRI-IACUC-099016-A) by the Institutional Animal Care and Use Committee (IACUC) of National Health Research Institutes (Zhunan, Miaoli County, Taiwan).

### High-content imaging and data processing

Cells were seeded in 96-well flat bottom plates (BD Biosciences) and co-transfected with *POU5F1* fluorescent reporter plasmid (*i.e.* pGL3-hOCT4_5 k-mCherry) and various combinations of expression plasmids of transcription factors and epigenetic regulators. After 48 hours of transfection, cells were imaged using a high-content imaging system (Molecular Devices). In each experimental condition, 25 images were collected with a 20× objective. Images were processed and fluorescence intensities were quantified using MetaMorph (Molecular Devices). Error analyses ± SD were obtained from triplicates of the experiments.

### RT-PCR

Total RNA was extracted from mouse neural stem cells using Illustra RNAspin Mini Isolation Kit (GE Healthcare). Complementary DNA (cDNA) was produced using the SuperScript III Reverse Transcriptase Kit (Invitrogen). Sequences of the primers to amplify mouse *Pou5f1* and *Neurod1* are (*Pou5f1*-F: 5′-CGTGAAGTTGGAGAAGGTGGAACCA; *Pou5f1*-R: 5′-CACCTCACACGGTTCTCAATGCTAGT; *Neurod1*-F: 5′-GGCAGACAAGAAAGAGGACG; *Neurod1*-R: 5′-GCGTCTGTACGAAGGAGACC). PCR product of mouse glyceraldehyde-3-phosphate dehydrogenase (*Gapdh*-F: 5′-ACCACAGTCCATGCCATCAC; *Gapdh*-R: 5′-TCCACCACCCTGTTGCTGTA) was served as an internal control. Real-time quantitative PCR (RT-qPCR) was performed using LightCycler TaqMan Master Kit (Roche) and Roche LightCycler System (Roche). Each PCR reaction contains 10–50 ng cDNA, 0.5 µM primer pairs and 0.1 µM Universal ProbeLibrary Probe (Roche). Gene expression levels were normalized to an internal control *Hprt1*. Primer sequences used for qPCR are (*Hprt1*-qPCR-F: 5′- TCCTCCTCAGACCGCTTTT; *Hprt1*-qPCR-R: CCTGGTTCATCATCGCTAATC; *Pou5f1*-qPCR-F: 5′-CTGGGCGTTCTCTTTGGA; *Pou5f1*-qPCR-R: 5′-GTTGTCGGCTTCCTCCAC; *Neurod1*-qPCR-F: 5′-CGCAGAAGGCAAGGTGTC; *Neurod1*-qPCR-R: 5′-TTTGGTCATGTTTCCACTTCC).

### Chromatin Immunoprecipitation (ChIP)-qPCR

Quantitative ChIP was performed according to the manufacturer's protocol (Millipore). In brief, mouse neural spheres treated without or with 5.0 mM valproic acid (VPA) for 48 hours were fixed with 1% formaldehyde for 10****minutes at room temperature, then 0.125 M Glycin was added to stop the reaction. DNA was sheared to ∼200–1000 bp using Bioruptor® (Diagenode). For each immunoprecipitation, chromatin from 1×10^6^ cells was precipitated with the indicated antibodies. Antibiodies against EP300, KDM5A, KDM6A and KDM6B were obtained from Abcam. For qPCR followed by ChIP, the LightCycler FastStart DNA Master^PLUS^ Kit (Roche) was used. Primer sequences used for qPCR of *Pou5f1* sequence within the proximal promoter (PP) [26] are [*Pou5f1*-PP-F(-205): 5′-GGTGAGAGGACCTTGAAGGTTGA; *Pou5f1*-PP-R(-4): 5′-CTAGGGACGGTTTCACCTCTCCC]. Primer sequences used for qPCR of *Pou5f1* CR2 region within the proximal enhancer (PE) [26] are [*Pou5f1*-PE-F(-945): 5′-GAGCCATCCTGGCCCATTCA; *Pou5f1*-PE-R (-755): 5′-CCCAGGCTTCCAGCCTAGTTC].

## Results

### HDACi promotes transcriptional activation in the upstream 5kb region of human *POU5F1*

Since Yamanaka and coworkers published their technique of deriving iPSCs from somatic cells using the four TFs (i.e. POU5F1, SOX2, KLF4 and MYC, OSKM) [3], [4], several variations on the original combination of ingredients (including TFs, small molecules, and cytokines) have been developed to improve the efficiency of induced pluripotency [27]. Of these, POU5F1 is currently the only non-replaceable factor for human iPSCs [2], [5], [6], [28]. Therefore, understanding the means by which transcription of *POU5F1* is regulated in ESCs and somatic lineages [17] is of paramount significance.

The 5.0 kb upstream region of human *POU5F1* (hOCT4_5 k) has been demonstrated to be functional in human embryonic stem cells, but inactive in human fibroblasts [4]. Histone acetylation neutralizes the positive charge in histones and reduces the interaction of histones and DNA, thereby relaxing the structure of chromatin and allowing access to the transcriptional machinery in the binding of DNA [18]. To determine whether histone acetylation influences hOCT4_5 k activity, HEK293T cells were transfected with pGL3-hOCT4_5 k-mCherry reporter plasmid for 24 hours. They were then treated using two HDAC inhibitors (HDACi), sodium butyrate (SB) and valproic acid (VPA) respectively, for an additional 24 hours. A pGL3-mCherry plasmid that did not contain a promoter sequence was used as a blank control (Figure 1A). The reporter activity was monitored using a high-content imaging system, and the fluorescence intensity was calculated using MetaMorph software. In HEK293T cells, hOCT4_5 k was silenced and therefore minimum fluorescence signal was observed. However, the treatment with the HDAC inhibitors enhanced hOCT4_5 k activity in a concentration-dependent manner (Figure 1B, C). Although HDAC inhibitors may non-specifically induce transcription of genes, these results demonstrate the feasibility of using this assay to determine factors that regulate the activity of hOCT4_5 k (Figure 1B, C) in HEK293T cells where hOCT4_5 k was silenced.

### Cooperation of NANOG, MYC and KLF4 for the transcriptional activation of hOCT4_5k

**Figure 2:**
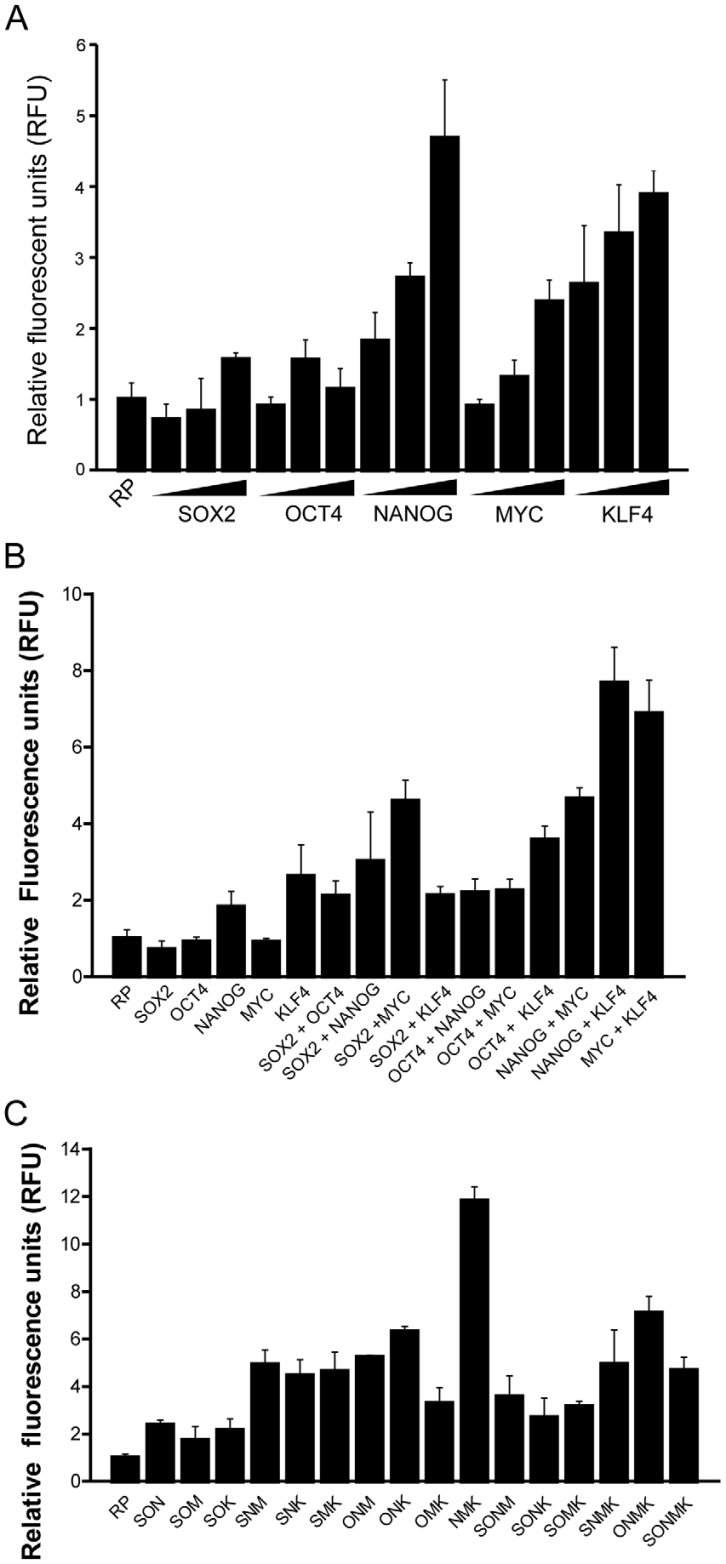
Combinatorial effect of the reprogramming-associated TFs in the transcriptional activation of hOCT4_5 k. (A) Activity of hOCT4_5 k in HEK293T cells overexpressed with increasing dosages of human SOX2, POU5F1, NANOG, MYC, or KLF4. (B-C) hOCT4_5k activity in cells overexpressed with 1–2 (B) or 3–5 (C) of the reprogramming-associated TFs. Cells were transfected with the minimum dosages of the TF-expression plasmids in (A). RP, pGL3-hOCT4_5k-mCherry reporter only; S, SOX2; O, POU5F1; N, NANOG; M, MYC; K, KLF4.

The iPSC technology demonstrates that terminally differentiated primary skin fibroblasts can be reprogrammed into ES-like cells using four TFs, OSKM [4]. In an independent study, NANOG has also been shown to be critically involved with induced pluripotency [7], [29]. Intriguingly, ectopically expressed TFs are eventually repressed due to retroviral silencing in ESCs [30], whereas cell-endogenous pluripotency-associated genes such as *POU5F1* and *NANOG* are activated. However, the means by which these TFs induce the transcription of endogenous pluripotent genes is less understood. To correlate the influence of the five reprogramming-associated TFs (i.e. POU5F1, SOX2, KLF4, MYC and NANOG, OSKMN) on hOCT4_5 k activity, we examined HEK293T cells co-transfected with pGL3-hOCT4_5 k-mCherry reporter and various quantities of the expression plasmids of human SOX2, POU5F1, MYC, NANOG, and KLF4, respectively. We determined that NANOG, MYC, and KLF4 significantly enhanced hOCT4–5k activity in a dose-dependent manner (Figure 2A). SOX2 was also shown to enhance hOCT4_5k activity, but its effect was relatively modest (Figure 2A) [15], [16], [31], [32].

To assess whether the reprogramming-associated TFs cooperate with one another to enhance hOCT4_5k activity, we co-transfected these five TFs using various combinations of two, three, four, or five factors, and subsequently examined the pGL3-hOCT4_5k-mCherry reporter activity (Figure 2B–C) [15], [31]. The results indicate that NANOG+KLF4, MYC+KLF4 (Figure 2B), and NANOG+MYC+KLF4 (Figure 2C) have a significant synergistic effect on the transcriptional activation of hOCT4_5k. We also noted that while SOX2 or POU5F1 alone did not perturb the activity of hOCT4_5k, the combinations of SOX2+POU5F1 [32], SOX2+MYC or POU5F1+MYC increased approximately 2–4 folds the activity of hOCT4_5k (Figure 2B). On the other hand, having four or five of the reprogramming-associated TFs did not further promote hOCT4_5k activity (Figure 2C). These results suggest the existence of a delicate stoichiometric mechanism between the reprogramming-associated TFs in regulating *POU5F1* expression.

### Identification of chromatin regulators that participate in the transcriptional activation of hOCT4_5k

**Figure 3:**
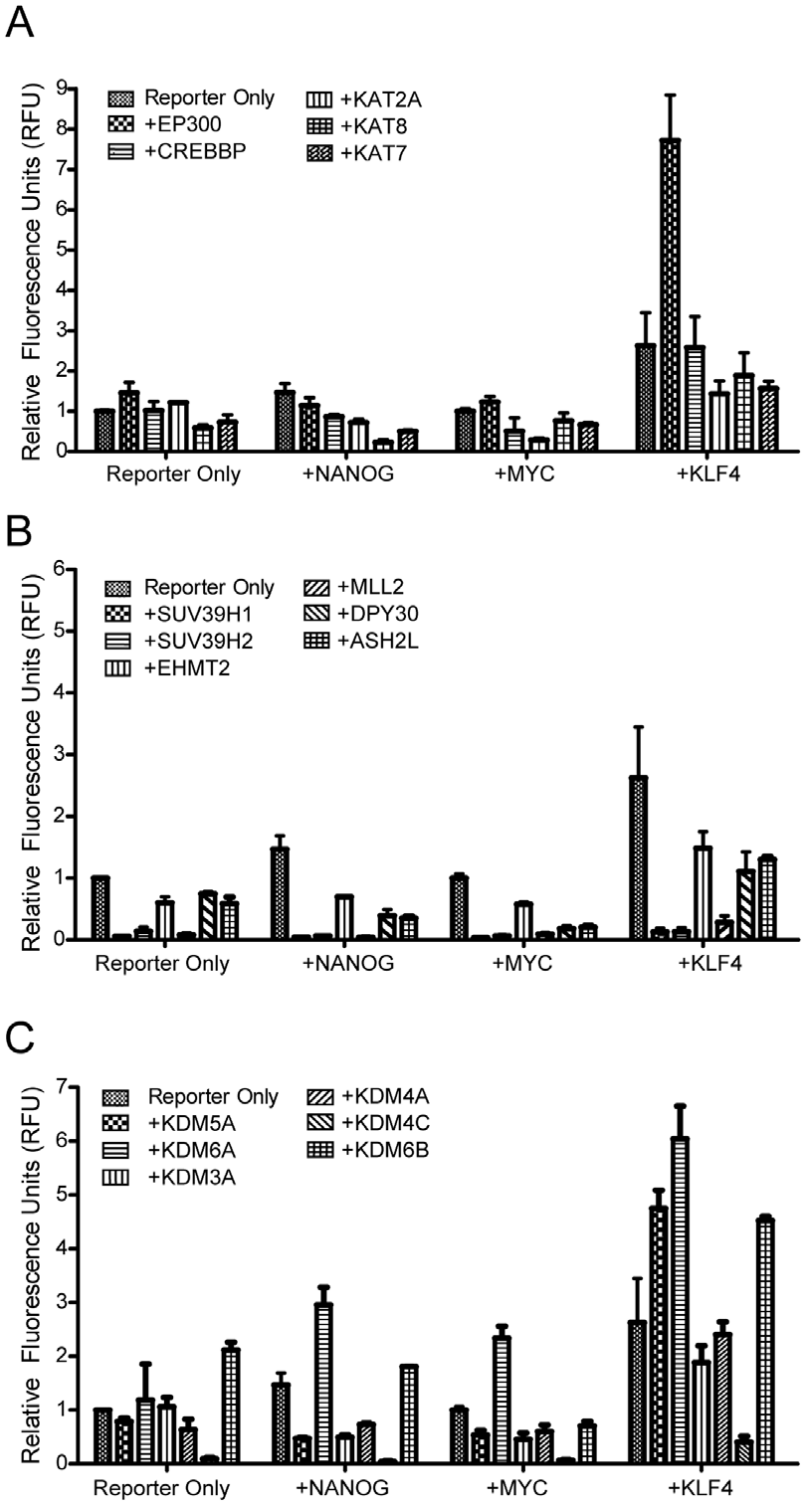
Crosstalk among the reprogramming-associated TFs and epigenetic modification enzymes in the activity of hOCT4_5k. The hOCT4_5k activity in HEK293T cells co-overexpressed with TFs NANOG, MYC or KLF4 and (A) histone acetyltransferases (EP300, CREBBP, KAT2A, KAT8, KAT7), (B) histone methyltransferases (SUV39H1, SUV39H2, EHMT2, MLL2, DPY30, ASH2L) or (C) histone demethylases (KDM5A, KDM6A, KDM3A, KDM4A, KDM4C, KDM6B).

Histone epigenetic modifications, including acetylation and methylation, have been shown to coordinate with cellular TFs in the regulation of genes [18]. The acetylation of histones neutralizes the electrostatic interaction between the histone tails and DNA, leading to the decompression of chromatin and the transcription of genes. To determine which histone acetyltransferase (HAT) participates in the transcriptional activation of hOCT4_5k, we examined the activity of pGL3-hOCT4_5k-mCherry reporter in HEK293T cells co-transfected with five HATs (EP300 and CREBBP from the CBP/P300 family, KAT2A from the GNAT family, and KAT8 and KAT7 from the MYST family), respectively. Among these, a slight (1.5 fold) increment in the activity of hOCT4_5k was observed in cells overexpressing EP300 (Figure 3A, left column), while no difference was observed in the hOCT4_5k activity in cells overexpressing the other four HATs.

In addition to acetylation, histone H3K4 methylation is frequently associated with gene activation. In contrast, both histone H3K9 methylation and histone H3K27 mehtylation are associated with gene repression [18]. To determine which histone methylation or demethylation regulator(s) contribute to the transcription of hOCT4_5k, pGL3-hOCT4_5k-mCherry reporter was co-transfected with histone methyltransferases (H3K9: SUV39H1, SUV39H2 and EHMT2; H3K4: MLL2, DPY30 and ASH2L; Figure 3B) or histone demethylases (H3K4: KDM5A; H3K27: KDM6A and KDM6B; H3K9: KDM3A and KDM4A; H3K36: KDM4C; Figure 3C), respectively. The fluorescence intensity was then measured. Introducing the six histone methyltransferases alone did not appear to enhance the activity of hOCT4_5k (Figure 3B, left column). In contrast, the histone H3K27 demethylase KDM6B increased pGL3-hOCT4_5k-mCherry reporter activity 2 fold (Figure 3C, left column).

To determine whether crosstalk existed between the TFs and the chromatin regulators in the enhancement of hOCT4_5k activity, a TF (NANOG, MYC or KLF4) expression vector was transfected together with a chromatin regulator, using pGL3-hOCT4_5k-mCherry reporter. As a result, the activity of hOCT4_5k was further promoted when KLF4 was co-expressed with EP300, KDM5A, KDM6A or KDM6B, respectively (Figure 3A–C). A slight increase in the activity of hOCT4_5k was also observed in HEK293T cells co-expressed with KDM6A and NANOG or MYC. Conversely, there is no synergistic effect in the activity of hOCT4_5k observed between histone methyltransferases and NANOG, MYC or KLF4. These data revealed that the histone acetyltransferase EP300 and the histone demethylases KDM5A, KDM6A and KDM6B, cooperate with KLF4 in the transcriptional activation of hOCT4_5k.

### HDAC inhibitors induce expression of *Pou5f1* in mouse neural stem cells

**Figure 4:**
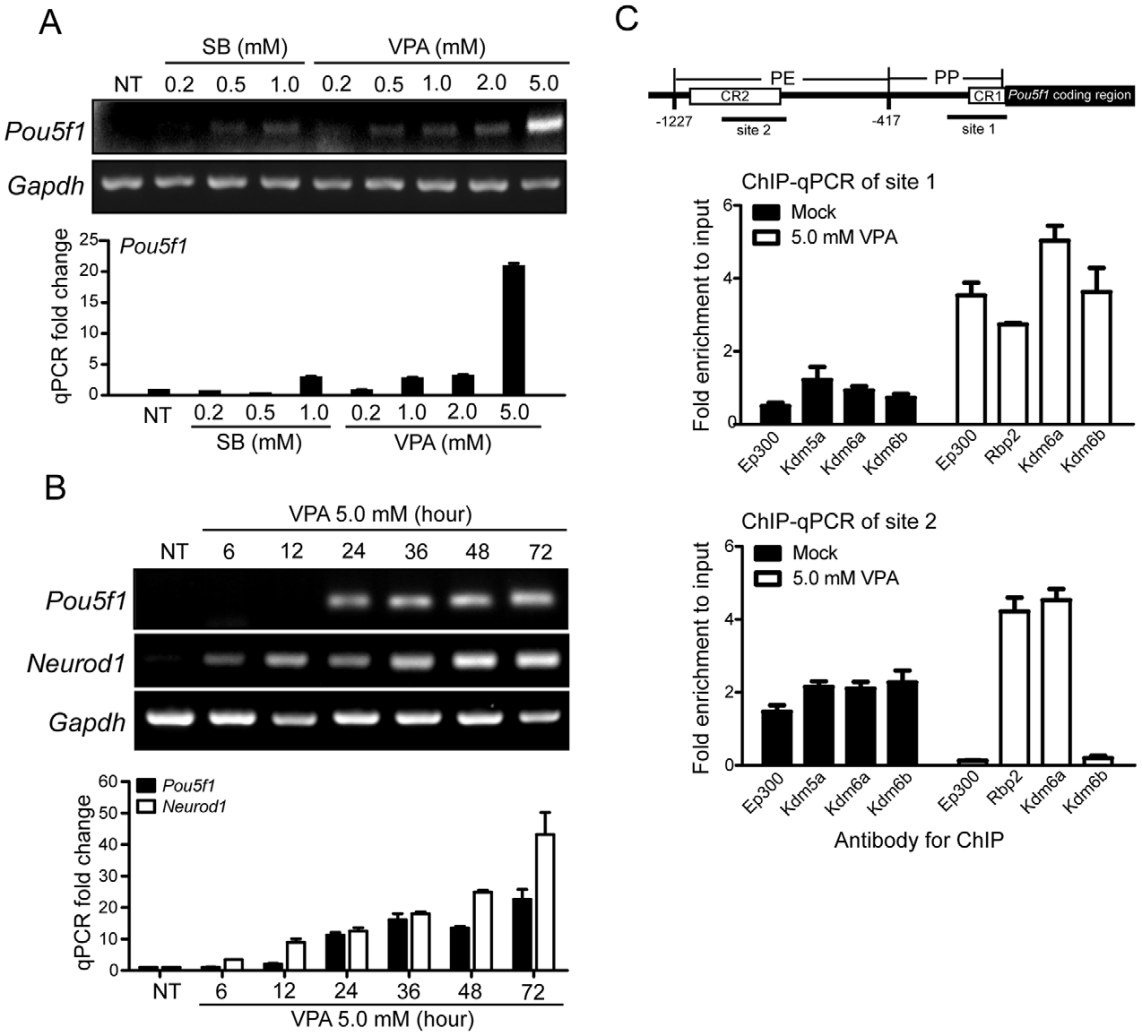
HDAC inhibitors transiently induce *Pou5f1* expression in mouse neural stem cells. (A) Expression of endogenous *Pou5f1* by RT-PCR and qRT-PCR (lower scheme) in mouse neurospheres treated with various concentrations of sodium butyrate (SB) or valproic acid (VPA) for the duration of 48 hours. (B) Time-dependent expression of endogenous *Pou5f1* and *Neurod1* by RT-PCR and qRT-PCR (lower scheme) in mouse neurospheres treated with 5.0 mM VPA. *Gapdh* was used as an internal quantitative control. NT, not treated. (C) (Upper) Scheme of mouse *Pou5f1* upstream sequence containing the proximal promoter (PP) and proximal enhancer (PE). (Lower) ChIP-qPCR of Ep300, Kdm5a, Kdm6a and Kdm6b at the indicated sites within PP and PE of *Pou5f1* in mouse neural stem cells treated with mock or 5.0 mM VPA for 48 hours. The amplification value from ChIP was normalized to 10% DNA input. Bars are averages±SD of triplicate experiments.

The above assays demonstrate that *POU5F1* transcription can be regulated epigenetically through cooperation of a subset of TFs and epigenetic regulators. Neural stem cells (NSCs) endogenously express Sox2, Myc, and Klf4 as well as several intermediate reprogramming markers [6], [33]. Based on the intrinsic gene expression profiles of NSCs and our results (Figures 1, 2, 3), we ask if *Pou5f1* expression can be induced in NSCs by reinforcing chromatin relaxation using HDAC inhibitors. *Pou5f1* expression was examined using RT-PCR and qRT-PCR in mouse embryonic NSCs treated with various concentrations of SB or VPA for 48 hours (Figure. 4A). Indeed, treatment with 1.0 mM SB or 2.0 mM VPA increased but modestly (∼3 fold as quantified by qRT-PCR, Fig. 4A, lower graph), the *Pou5f1* expression, while treatment with 5.0 mM VPA increased 20 fold *Pou5f1* expression in mouse NSC (Figure. 4A).

The effect of HDACi in cell self-renewal and lineage commitment appears to be biphasic [34]. Although HDACi enhances efficiency in the generation of iPSCs from primary human fibroblasts, HDACi has also been shown to induce neuronal differentiation by up regulating neuronal-specific genes such as *Tubb3*, *Neurod1*, and *Neurog1* [35], [36]. To determine the temporal effect of HDACi in mouse NSC lineage specifications, we examined expression of *Pou5f1* and a neuronal marker *Neurod1* in mouse NSC treated with VPA (5.0 mM) for various durations. Intriguingly, treatment with VPA induced expression of *Pou5f1* within 12 hours post treatment (∼2 fold), and reached a plateau at 36 hours (∼20 fold) post treatment. Conversely, the expression of *Neurod1* kept increasing (Figure. 4B), which is an indication of neuronal commitment. These data suggest the histone acetylation-induced *Pou5f1* transcription can be achieved within 12 hours by VPA in mouse NSCs. These results also demonstrate the spatial and temporal regulation of lineage-specific genes by VPA in mouse NSCs.

In order to understand the functional relevance of the epigenetic regulators in *Pou5f1* expression (Figure 3), using chromatin immunoprecipitation (ChIP), we compared interaction of Ep300, Kdm5a, Kdm6a and Kdm6b to *Pou5f1* proximal promoter (PP) and proximal enhancer (PE) [26] in mouse neural stem cells treated without or with 5.0 mM VPA (Figure 4C). Our results show that there is enrichment of Ep300 and Kdm6b at PP but not PE of *Pou5f1* upon VPA treatment. On the other hand, VPA provoked increased recruitment of Kdm5a and Kdm6a to both PP and PE of *Pou5f1*. These results demonstrate Kdm5a and Kdm6a may specifically contribute to *Pou5f1* activation by interacting with both PP and PE of its promoter.

## Discussion

While the mechanism underlying reprogramming remains enigmatic, it is clear that induced pluripotency can be achieved from multiple cellular lineages [2], [13], [28], [37]. Amongst these cells, mammalian NSCs can be reprogrammed to become iPSCs by a single transcription factor, POU5F1 [6], [37]. The result of one-factor reprogramming suggests that NSCs which endogenously express several reprogramming-associated TFs, Sox2, Klf4 and Myc, may represent an intermediate state between differentiated and pluripotent cells [6], [37]. POU5F1, which is non-replaceable in current reprogramming methods, has attracted substantial attentions for studies in its gene regulatory mechanisms [14]–[17]. Using murine NSCs, in this study we showed that VPA alone is sufficient to induce endogenous *Pou5f1* expression within 12 hours of treatment (Figure 4B). However, prolonged treatment of VPA activates transcription of neuronal genes and subsequent cellular differentiation towards the neuronal lineage. These data suggest that the *Pou5f1* gene may be positioned in a chromatin region that is less compact for it to respond to VPA prior to neuronal genes in mouse NSCs. This finding also supports the notion that deacetylation of histones may be the major epigenetic factor responsible for *Pou5f1* silencing in mouse NSCs. Because Pou5f1 is the only factor that needs to be supplemented exogenously to convert NSCs into iPSCs, and because *Pou5f1* expression can be induced by VPA in mouse NSCs, it may be achievable to generate induced pluripotency from NSCs simply by VPA and appropriate culture conditions.

Despite the success of iPSC, it remains unclear how the exogenously overexpressed TFs regulate expression of endogenous pluripotent genes such as *POU5F1*. To identify epigenetic regulators that may participate in *POU5F1* gene expression, we profiled the crosstalk between 5 reprogramming-associated TFs and 17 chromatin regulators for transcriptional activation in the upstream 5.0 kb region of human *POU5F1* in HEK293T cells, where *POU5F1* is silenced (Figures 2–3). We identified the stoichiochemical relationship of the 5 reprogramming-associated TFs, POU5F1, SOX2, NANOG, MYC and KLF4, for *POU5F1* transcription (Figure 2). We also discovered that the histone acetyltransferase EP300, the histone demethylases KDM5A, KDM6A and KDM6B, cooperate with KLF4 in the transcriptional activation of *POU5F1* (Figure 3). In our results (Figure 2), although the combination of NANOG+KLF4 and NANOG+KLF4+MYC induces *POU5F1* activity to ∼8-folds and ∼12-folds, respectively, adding more factors (such as SNMK or ONMK) does not always contribute to *POU5F1* activation. This fluctuation may be attributed to cell-to-cell extrinsic heterogeneity or the inherent stochastic nature of gene expression or regulatory signaling processes [38]. Indeed, a negative feedback loop composed by FoxD3-Nanog-Pou5f1 has been reported to regulate the expression of *Pou5f1* for self-renewal [39]. Moreover, when POU5F1 is expressed two-fold, it induces differentiation into primitive endoderm and mesoderm, whereas a 50% decrease in POU5F1 causes differentiation into trophectoderm [40]. Thus, delicate stoichiometric regulation is required to control the level of *POU5F1* expression for self-renewal and pluripotency in ESCs.

Of the four chromatin regulators that we identified as cooperating with KLF4 in transcriptional activation of *POU5F1*, the H3K27 demethylase KDM6A is recently demonstrated to regulate somatic and germ cell epigenetic reprogramming [41]. Although KDM6A ectopic overexpression in addition to OSKM does not lead to increased efficiency in iPSC formation, neither is it required for pluripotent state maintenance, KDM6A is essential for the induction of the pluripotent state at the transcriptional level during in vitro reprogramming through its H3K27 demethylation activity [41]. Kdm6a has also been found to associate with Pou5f1, Klf4 and Sox2 by a protein-protein interaction and promotes the reactivation of potent pluripotency promoting modules that cooperatively facilitate iPSC formation [41]. In addition, ChIP-sequencing analysis showed that Kdm6a specifically bound 1,845 target genes including *Pou5f1* and *Nanog* in mouse embryonic fibroblasts [41]. Our new findings in the enrichment of Kdm6a at PP and PE of *Pou5f1* promoter in mouse NSCs upon VPA treatment (Figure 4C) and the cooperative role for Kdm6a and KLF4 in *POU5F1* transcriptional activation (Figure 3C) supports their results [41], and may also explain, at least in part, the molecular role of Kdm6a in inducted pluripotency.

In addition to KDM6A, of the three other epigenetic regulators that we identified, the histone acetyltransferase EP300 has been shown to play an important role in the differentiation process of ESCs [42]. Ep300 has also been demonstrated to interact with Klf4 and regulate gene transcription by modulating histone acetylation [43]. KDM6B demethylates tri-methylated H3K27, and its ectopic expression causes delocalization of polycomb proteins in vivo [23], [44]. In addition, inhibiting the expression of a Kdm6b orthologue in *C. elegans* results in the development of abnormal gonads [44]. KDM5A belongs to a demethylase family specific to tri-and dimethylated H3K4 [45] and is associated with a large number of PcG (Polycomb group) target genes in mouse ESCs [46]. The recruitment of KDM5A to Polycomb-repressive complex 2 (PRC2) is normally associated with repressive activity during ES cell differentiation [46]. Intriguingly, increased expression of KDM5A was found in hatched blastocysts during early porcine embryonic development [47]. In our hands, over expression of KDM5A alone did not affect hOCT4_5k activity (Fig. 3C). Therefore, the cooperative effect between KLF4 and KDM5A may be specific to hOCT4_5k activity. In mouse neural stem cells, KDM5A was recruited to PP and PE of *Pou5f1* upon VPA treatment (Figure 4C). These results suggest KDM5A may play a role in generating bivalent chromatin structure marks [48] for the delicate stoichiometric regulation of *Pou5f1* expression. Whether EP300, KDM5A or KDM6B contribute directly or indirectly to induced pluripotency or lineage commitment in the primary cell system warrants future investigation. Nevertheless, the assay designed in this study provides a strategy to discover new epigenetic regulators that activate POU5F1 transcription. With the growth of understanding regarding genes and culture conditions associated with the interchange between pluripotency and lineage commitment [10], [27], [37], the development of clinically applicable methods for reprogramming from various cell types is anticipated in the near future.
